# Reference genome for the Mojave poppy bee (*Perdita meconis*), a specialist pollinator of conservation concern

**DOI:** 10.1093/jhered/esad076

**Published:** 2023-12-13

**Authors:** Rena M Schweizer, Colleen G Meidt, Ligia R Benavides, Joseph S Wilson, Terry L Griswold, Sheina B Sim, Scott M Geib, Michael G Branstetter

**Affiliations:** U.S. Department of Agriculture, Agricultural Research Service (USDA-ARS), Pollinating Insects Research Unit, Utah State University, Logan, UT, United States; Division of Biological Sciences, University of Montana, Missoula, MT, United States; U.S. Department of Agriculture, Agricultural Research Service (USDA-ARS), Pollinating Insects Research Unit, Utah State University, Logan, UT, United States; Department of Biology, Utah State University, Logan, UT, United States; U.S. Department of Agriculture, Agricultural Research Service (USDA-ARS), Pollinating Insects Research Unit, Utah State University, Logan, UT, United States; Department of Biology, Utah State University—Tooele, Tooele, UT, United States; U.S. Department of Agriculture, Agricultural Research Service (USDA-ARS), Pollinating Insects Research Unit, Utah State University, Logan, UT, United States; U.S. Department of Agriculture, Agricultural Research Service, U.S. Pacific Basin Agricultural Research Center, Tropical Pest Genetics and Molecular Biology Research Unit, Hilo, HI, United States; U.S. Department of Agriculture, Agricultural Research Service, U.S. Pacific Basin Agricultural Research Center, Tropical Pest Genetics and Molecular Biology Research Unit, Hilo, HI, United States; U.S. Department of Agriculture, Agricultural Research Service (USDA-ARS), Pollinating Insects Research Unit, Utah State University, Logan, UT, United States

**Keywords:** Andrenidae, conservation genetics, PacBio HiFi, repetitive elements

## Abstract

The Mojave poppy bee, *Perdita meconis* Griswold (Hymenoptera: Anthophila: Andrenidae), is a species of conservation concern that is restricted to the eastern Mojave Desert of North America. It is a specialist pollinator of two poppy genera, *Arctomecon* and *Argemone* (Papaveraceae), and is being considered for listing under the US Endangered Species Act along with one of its pollinator hosts, the Las Vegas bearpoppy (*Arctomecon californica*). Here, we present a near chromosome-level genome of the Mojave poppy bee to provide a genomic resource that will aid conservation efforts and future research. We isolated DNA from a single, small (<7 mm), male specimen collected using non-ideal preservation methods and then performed whole-genome sequencing using PacBio HiFi technology. After quality and contaminant filtering, the final draft genome assembly is 327 Mb, with an N50 length of 17.5 Mb. Annotated repetitive elements compose 37.3% of the genome, although a large proportion (24.87%) of those are unclassified repeats. Additionally, we annotated 18,245 protein-coding genes and 19,433 transcripts. This genome represents one of only a few genomes from the large bee family Andrenidae and one of only a few genomes for pollinator specialists. We highlight both the potential of this genome as a resource for future research, and how high-quality genomes generated from small, non-ideal (in terms of preservation) specimens could facilitate biodiversity genomics.

## Introduction

Wild bees are critical to the health and productivity of the world’s natural and agricultural systems (Patel et al. 2021), yet many bees are declining due to disturbance, pathogens, habitat loss, climate change, and other factors still unknown (Archer et al. 2014). There are over 20,000 species of bees in the world, with 3,594 of them described in the United States alone (discoverlife.org; last accessed 28 August 2023). Among this diversity, only 12 bee species are managed for crop pollination globally (Patel et al. 2021); however, evidence suggests that the remaining tens of thousands of wild, unmanaged bee species likely contribute more significantly to crop pollination than managed pollinators (Kleijn et al. 2015). Much of the health and productivity of an ecosystem, both in managed and wild areas, depends on the complementarity of wild bee species (Blitzer et al. 2016), yet the role and importance of wild bee species is not well understood. Thus, there is an increased and urgent interest in studying wild species to ensure a future with humans and the sustained health of our planet. This call to action necessitates adequate tools for describing and monitoring bees.

Population genetics at a genomic scale is one such tool that has revolutionized our ability to understand and conserve species through determining key population parameters (e.g. effective population size, genetic diversity, heterozygosity; Allendorf et al. 2010; Allendorf 2017), discovering evolutionarily distinct units (e.g. Funk et al. 2012), characterizing inbreeding depression (Kardos et al. 2016) and informing genetic rescue (Bell et al. 2019). A large number of genome-wide markers enables more precise estimates of traditional conservation genetic parameters and allows quantification of a wider range of adaptive and neutral factors (Allendorf et al. 2010) in a rigorous and powerful manner (Formenti et al. 2022). Reference genomes can also be used to investigate genomic aspects of insect health (e.g. Grozinger and Zayed 2020; Chua et al. 2023), the genetic basis and demographic history of specialization (e.g. Pope et al. 2023), and more, expanding the kinds of comparative analyses that can be done on rare or threatened species. Recent advances in sequencing technologies and concurrent reduction in costs have enabled a new era of insect genomics, with additional genomes on the horizon (e.g. Hotaling et al. 2020, 2021; Woodard et al. 2020; Childers et al. 2021). Here we present a reference genome for the important wild pollinator species *Perdita meconis* Griswold.

**Fig. 1.**
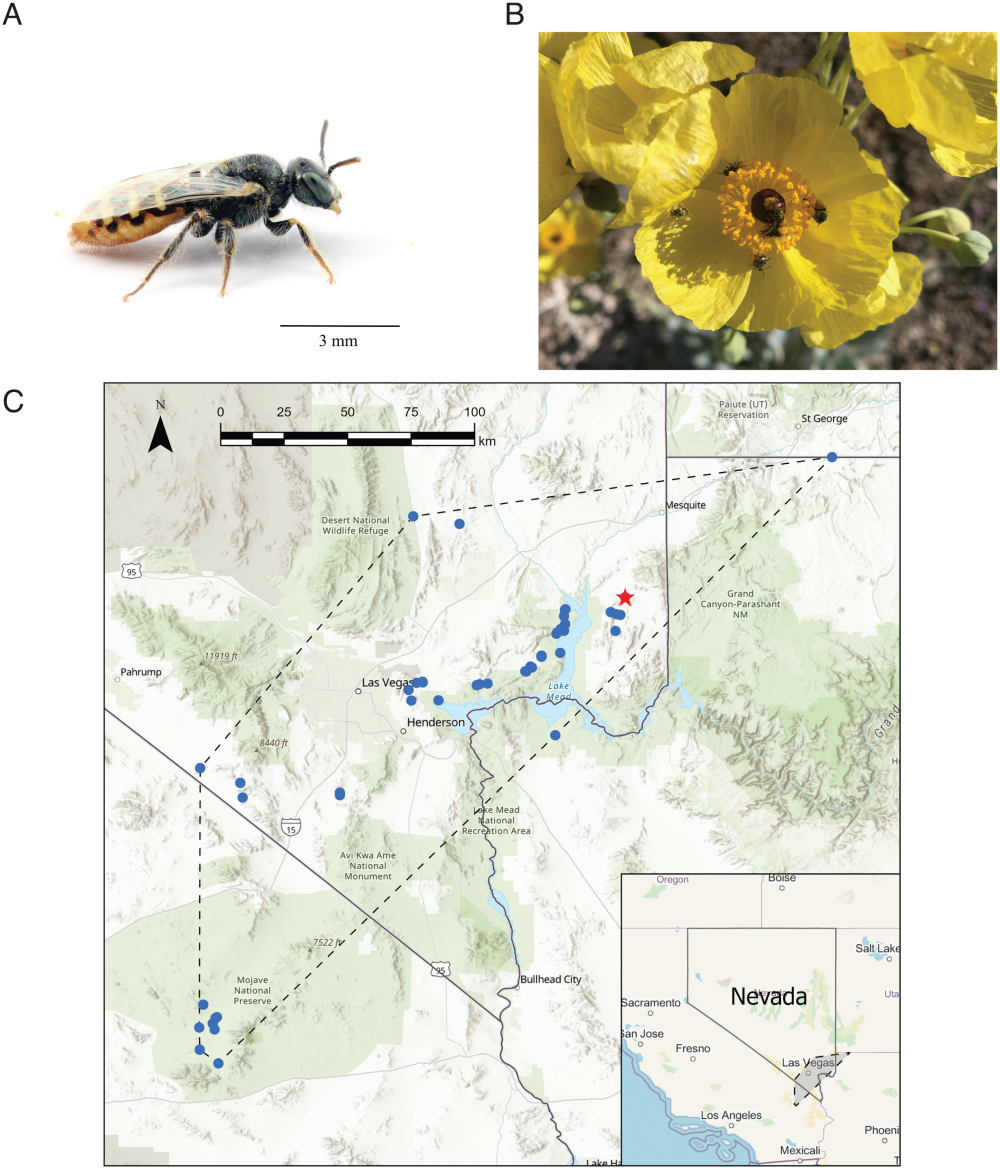
Specimen information for the Mojave poppy bee (*Perdita meconis*), a species of conservation concern. A) Individuals of this rare species are 5 to 7 mm in body length and B) specialize on only a few plant species including Las Vegas bearpoppy (*Arctomecon californica*), pictured here with multiple *Perdita* mating while foraging for pollen. C) The sampling distribution representing the entire range of the Mojave poppy bee based on surveys conducted by the USDA. Dots (blue) indicate sampling locations where the Mojave poppy bee was present, while the star (red) indicates the sampling location of the specimen used for genome sequencing. Photographs by Joe Wilson (A) and Colleen Meidt (B).

*Perdita* Smith 1853 (Hymenoptera: Andrenidae: Panurginae) is the most species-rich bee genus in North America, encompassing approximately 640 described species concentrated in xeric regions of the United States and Mexico (Portman and Griswold 2017). It is a member of the family Andrenidae, commonly referred to as “mining bees” for their ground nesting behavior (Michener 2007). Most species of *Perdita* are small in size and are host-plant specialists (Portman and Griswold 2017), meaning that they gather pollen from only a few closely related species of plants (Zayed et al. 2005). An exemplar species from this group is *P. meconis,* or the Mojave poppy bee (Fig. 1A), first discovered in Washington County, Utah, in 1988 (Griswold 1993). *P. meconis* is endemic to the eastern portion of the Mojave Desert in the United States and is restricted primarily to patchy habitats of cryptobiotic gypsum soils. The species is also an extreme specialist of only a few plant species in the poppy genera *Arctomecon* and *Argemone* (Fig. 1B). The bee is small, with a body length of 5 to 7 mm, and is suspected to have a limited dispersal range (Greenleaf et al. 2007).

Along with its host plants, *P. meconis* is of high conservation concern (Schwarzbach and Kadereit 1999; Portman et al. 2018); historically, its distribution spanned southeastern California, southern Nevada, northwestern Arizona, and southwestern Utah (Fig. 1C). However, within the last few decades, evidence suggests that *P. meconis* and its host plants have declined in their distributions. Recent surveys have failed to detect the bee from sites around St. George, Utah, likely due to habitat loss and human development (Portman et al. 2018). Although very little is known about this bee, studies suggest *P. meconis* has a direct impact on the survival and health of its host plants as it historically has been one of the most dominant floral visitors (Stosich et al. 2022). *Arctomecon humilis*, which occurs at the Utah sites where *P. meconis* used to be found, is listed as federally protected under the US Endangered Species Act. Surveys of *Arctomecon californica* have indicated poppy populations have declined and the species’ range has been reduced by half within the last 30 years (Stosich et al. 2022). Because of these declines, both *A. californica* and *P. meconis* are currently under review to be federally protected (U.S. Fish and Wildlife Service 2019).

To characterize *P. meconis* and to better inform conservation decisions within this pollinator system, we are sequencing genomic data from field-collected specimens. Here we present a highly contiguous sequence of *P. meconis*, the first published genome assembly of a bee in the genus *Perdita*, and one of few Andrenidae genomes. In addition to providing an initial peek into the genome of an ecologically important yet threatened bee species, this reference genome is an important starting point for future comparative and population genomic studies.

## Methods

### Specimen collection, DNA isolation, and quality control

In May 2020, we collected a series of individuals of *Perdita* from *A. californica* plants at Gold Butte National Monument, NV (36.50°N, 114.19°W). The specimens were collected alive and chilled in a cooler for several hours and then warmed up to room temperature for photographing by one of the authors (JSW). Following photography, the specimens were freeze-killed and stored in a standard −20 °C freezer until transferred to the USDA lab where they were stored in a −80 °C lab freezer. We report these details about specimen handling because freeze-killing specimens at −20 °C is generally suboptimal for generating ultra-high-molecular weight DNA for long-read sequencing (Dahn et al. 2022). Specimens were later viewed on ice under a dissecting microscope and several male and female specimens of *P. meconis* were expertly identified by one of us (TLG). Two male specimens were then shipped on dry ice to the USDA lab in Hilo, HI, where they were extracted and prepared for sequencing.

Given the small size of *P. meconis* specimens (5 to 7 mm), we used the entire body of a single haploid male for high-molecular weight DNA extraction. We used the MagAttract HMW DNA kit (Qiagen, Hilden, Germany) and followed the manufacturer’s protocol. Following extraction, we performed a 2× SPRI-bead cleanup for further purification and sample concentration. We measured DNA quantity and purity using a DS-11 Spectrophotometer/Fluorometer (Denovix, Wilmington, Delaware, USA) with Qubit High Sensitivity reagents (ThermoFisher Scientific, Waltham, Massachusetts, USA). DNA was sized on a Femto Pulse capillary electrophoresis system (Agilent, Santa Clara, California, USA).

A female specimen from the collection series (same sampling time and location) has been imaged (Fig. 1A) and another specimen from the same series has been deposited into the US National Pollinating Insects Collection as a voucher for this genome (NPIC; unique specimen identifier#: BBSL1182693).

### PacBio HiFi library preparation

From the entire DNA extraction, we prepared a single SMRTbell library using the SMRTbell library preparation kit (PacBio, San Diego, California, USA) following manufacturer’s protocols for low-input samples. DNA was sheared to 15 to 20 kb using a Megaruptor 2 (Diagenode, Denville, New Jersey, USA) prior to library preparation, and the final library was sized with Ampure PB beads (Beckman Coulter Life Sciences, Indianapolis, Indiana, USA) to remove fragments less than 3 kb in length. To prevent sample loss, no additional sizing was performed. The entire library was quantified using Qubit HS DNA reagents, sized on an Agilent Fragment Analyzer to determine molar concentration, and then bound and loaded for sequencing on a single 8M cell on a PacBio Sequel IIe system at the USDA Meat Animal Research Center in Clay Center, NE. The resulting subreads were used to generate circular consensus sequences on-instrument, generating HiFi read data used for assembly.

### Nuclear genome assembly

From the HiFi read data, we filtered out adapter contamination using HiFiAdapterFilt v. 0.2.3 (Table 1; Sim et al. 2022) with default parameters, then assessed quality metrics of filtered reads using fastqc v. 0.11.9 (https://www.bioinformatics.babraham.ac.uk/projects/fastqc/). Next, we performed genome assembly using HiFiASM v. 0.16.1-r375 (Cheng et al. 2021) with the command “hifiasm --n-hap 1” to denote the expected ploidy and supplied with the output of HiFiAdapterFilt in a compressed.fastq format. We refer to this output from HiFiASM as our “initial assembly.”

**Table 1.** Software information and assembly workflow for *Perdita meconis* assembly. Software citations are in the text.

Workflow step	Software and options	Version
Filtering PacBio HiFi adapters	HiFiAdapterFilt	0.2.3
Sequencing quality assessment	fastqc	0.11.9
K-mer counting	Meryl (*k* = 21)	1
Estimation of genome size and coverage	GenomeScope	2
De novo assembly	HiFiasm (--n-hap 1)	0.16.1-r375
Genome quality assessment		
Basic assembly metrics	BBMap	38.90
Assembly completeness	BUSCO (-m geno)	5.0.0
Contamination screening		
Local alignment tool	BLAST+	2.13.0
General contamination screening	BlobToolKit	2.6.1
Repeat analysis		
Identification of LTR elements	RepeatModeler (ltrstruct)	2
Annotation of TE diversity	RepeatMasker	4.1.2
Annotation		
Gene prediction	Braker	3
Gene annotation	Interproscan	5.60-92.0

### Mitochondrial genome assembly

We identified and extracted the mitochondrial genome using MitoHiFi v2 (Uliano-Silva et al. 2023), and annotated the mitogenome using Mitos (Bernt et al. 2013). MitoHiFi’s findMitoReference identified the mitochondrial genome of *Andrena chekiangensis* (NCBI accession NC_042768.1) as the closest reference genome to be used to guide the mitochondrial genome assembly and gene annotation.

### Genome assembly assessment of quality and continuity

We assessed genome quality and completeness using the BlobToolKit (Challis et al. 2020), which characterizes contig lengths (e.g. N50, N90) and assigns contigs to their closest taxon using BLAST (Altschul et al. 1997) and Diamond (Buchfink et al. 2015) searches to NCBI nt and UniProt databases, respectively. We also used the Benchmark of Universal Single-Copy Orthologs (BUSCOs; Waterhouse et al. 2017) within BlobToolKit, using relevant taxonomic databases for *P. meconis* (i.e. Eukaryota, Metazoa, Insecta, Endopterygota, and Hymenoptera). Additionally, we ran GenomeScope2 (Ranallo-Benavidez et al. 2020) to estimate genome size and coverage, and KAT to assess k-mer frequencies (Mapleson et al. 2017). We removed from the main assembly all contigs classified as non-arthropod by the BlobToolKit taxonomic identification above. The main assembly with non-arthropod contigs and mitogenome removed is referred to as the “draft genome assembly.”

### Chromosome identification

Given that we did not obtain HiC scaffolding data for *P. meconis*, we used alignment to chromosome-level assemblies of other bee species to identify contiguity of chromosome structure. Specifically, using nucmer within MUMmer4 (Marçais et al. 2018), we aligned the largest 22 contigs of the *P. meconis* genome (accounting for 90% of the total genome size) to the *Andrena camellia* (NCBI GCA_029448645.1) and *Apis mellifera* (NCBI GCF_003254395.2) genomes using default parameters. We visualized the genome-genome alignments using Dot (https://dot.sandbox.bio/).

### Repetitive content identification

A key step prior to gene annotation is to identify, then mask, repetitive regions in the genome. Thus, we used RepeatModeler v2.0.4 (Flynn et al. 2020) followed by RepeatMasker v4.1.5 (Smit et al.; http://www.repeatmasker.org/) to identify de novo transposable elements (TEs) and to soft mask *de novo* and known TEs from the draft genome assembly, respectively. Both of these software packages are included in the containerized version offered by the Dfam consortium (https://github.com/Dfam-consortium/TETools/; Storer et al. 2021).

### Gene annotation

To train and predict gene annotation of the draft genome assembly, we used BRAKER3 (Brůna et al. 2021) to fully automate the process. Given that we do not have RNAseq data for *P. meconis*, we implemented a BRAKER3 workflow (called pipeline C) that predicts genes with GeneMark-ETP and AUGUSTUS, using orthologous protein databases for Arthropods provided by OrthoDB v11 (Kuznetsov et al. 2022). We performed functional gene annotation using the Interproscan software v5.60-92.0 (Jones et al. 2014; Blum et al. 2020). These annotation methods follow previous studies on bee genomes without supporting transcriptome data (e.g. Sless et al. 2022).

### Phylogenomic placement of P. meconis

We constructed a phylogeny of bees and close relatives from within the superfamily Apoidea using Ultra Conserved Elements (UCEs) extracted from the iyPerMec1 draft genome assembly and other genomes, as follows. We downloaded all publicly available bee genomes from NCBI using the NCBI Datasets command line tools v. 15.6.0 (download date: 18 June 2023), then pruned the initial set of genomes to remove haplotype and species duplicates. To extract UCE loci from the genomes, we used Phyluce v. 1.7.1 (Faircloth 2016) and closely followed “tutorial 3” from the Phyluce documentation (https://phyluce.readthedocs.io/en/latest/tutorials/tutorial-3.html). UCEs were extracted by aligning the principal v2 UCE probe set for Hymenoptera from Branstetter et al. (2017) to the target genomes and by slicing out 500 bp of flanking sequence from either side of each UCE locus. For the probe alignment step, we set both the “min-coverage” and “min-identity” parameters to 80%.

Following UCE extraction, we used Phyluce to create a concatenated UCE matrix for phylogenetic analysis. We ran the phyluce_assembly_match_contigs_to_probes script using the bee-ant version of the probe set (described in Grab et al. 2019) and with the “min-coverage” and “min-identity” parameters set to 70% and 75%, respectively. We then aligned each locus with MAFFT v. 7.475 (Katoh and Standley 2013), trimmed the alignments with Gblocks v0.91b (Talavera and Castresana 2007) using reduced stringency parameters (b1 = 0.5, b2 = 0.5, b3 = 12, b4 = 7), and filtered loci to have 75% taxon completeness. We concatenated the alignments and inferred a phylogenetic tree using IQTree v. 2.2 (Minh et al. 2020) and a general time reversible model with gamma among site rate variation (GTR + F + G4). To measure support, we conducted 1,000 ultrafast bootstrap replicates (UFB; Hoang et al. 2018).

## Results

### Genome sequencing data

We sequenced DNA from a single haploid male individual of *P. meconis*, using PacBio HiFi sequencing obtaining 2,283,576 raw reads (17.13 Gb of raw data), 2,277,536 (99.7% of reads and 99.2% of data, 17.00 Gb) of which were retained after filtering.

### Initial genome assembly metrics

**Fig. 2.**
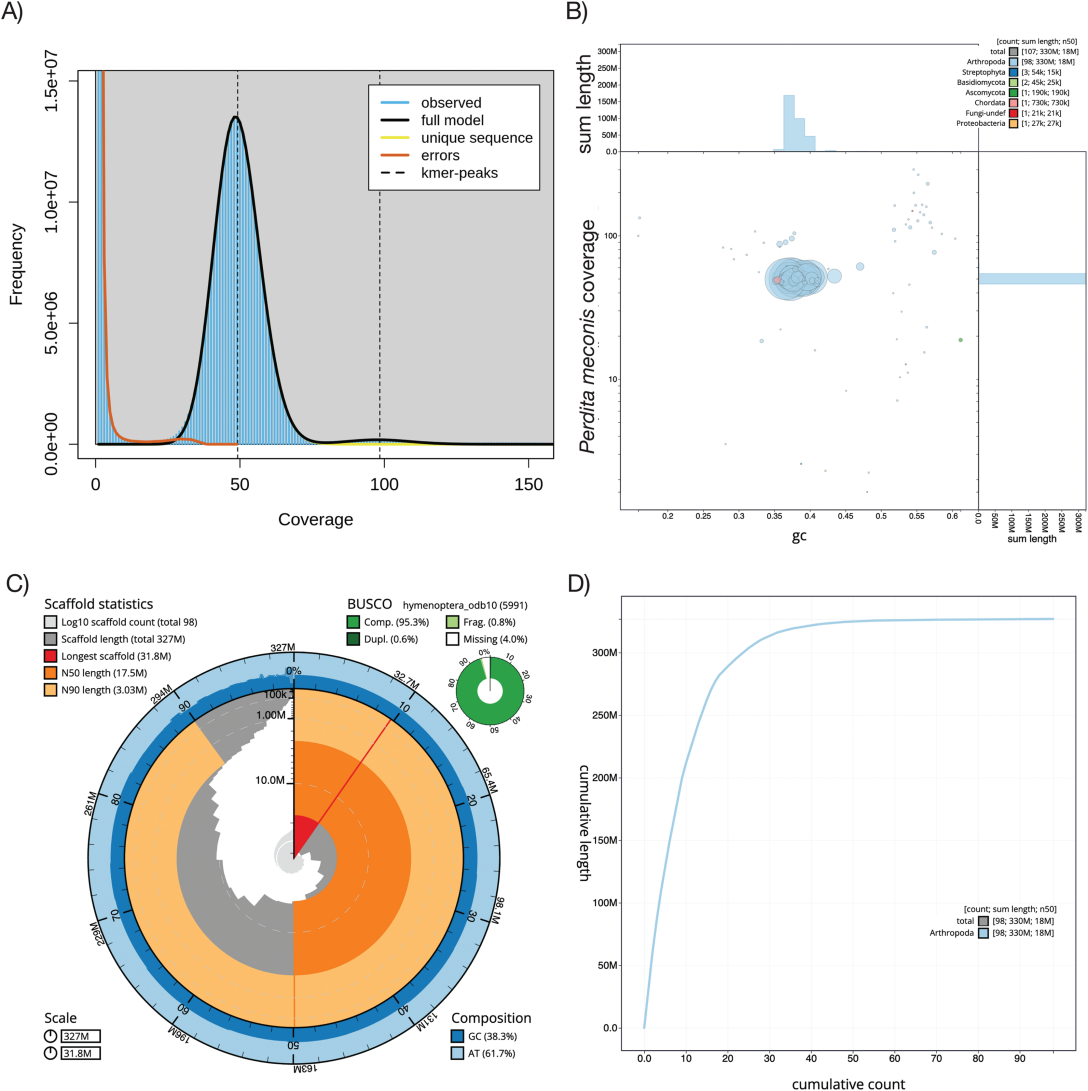
Genome assembly and quality metrics for the Mojave poppy bee genome. A) GenomeScope2 plot of k-mer frequency at varying depth of coverage for the initial assembly B) Blob plot for Mojave poppy bee initial genome assembly. Each blob represents a contig, colored according to the phylum with the best match using BLAST hits to the NCBI nucleotide database or Diamond BLAST hits to the Uniprot protein database. Most blobs are assigned to Arthropoda, with only a few assigned to fungi, plants, or Chordata. C) BlobToolKit snail plot of the final draft genome assembly after removal of non-arthropod contigs. The main plot is divided into 1,000 size-ordered bins around the circumference with each bin representing 0.1% of the 327,936,261 bp assembly. The distribution of sequence lengths is shown in dark gray with the plot radius scaled to the longest sequence present in the assembly (31,838,323 bp, shown in red). Orange and pale-orange arcs show the N50 and N90 sequence lengths (17,524,680 and 3,032,719 bp), respectively. The pale gray spiral shows the cumulative sequence count on a log scale with white scale lines showing successive orders of magnitude. The blue and pale-blue area around the outside of the plot shows the distribution of GC, AT, and N percentages in the same bins as the inner plot. A summary of complete, fragmented, duplicated, and missing BUSCO genes in the hymenoptera_odb10 set is shown in the top right. D) Plot of cumulative sequence length covered by contigs. The gray line shows cumulative length for all contigs, while the blue line shows cumulative length for Arthropoda contigs. In the final draft genome assembly with non-arthropod contigs removed, the blue and gray lines overlap.

The initial genome assembly is 327,936,261 bp, with an average depth of coverage of 49.2× as estimated by GenomeScope2 k-mer analysis (Table 1; Fig. 2A). The initial assembly consists of 107 gapless contigs, with a contig N50 length of 17.5 Mb, and an N90 length of 3.03 Mb (Supplementary Fig. S1). The longest contig is 31.8 Mb, with several others larger than 20 Mb, suggesting several full or near full chromosome contigs. Genome-wide mean GC content is 38.3% (Fig. 2B and C).

BUSCO analysis of the initial HiFi assembly found that 95.5% of the 5,991 Hymenoptera genes (in OrthoDB v10 Hymenoptera reference set) were complete, 0.8% were fragmented, 3.8% were missing, and 0.6% were duplicated (Fig. 2C).

### Non-arthropod taxonomic assignment

Using the Blobtools/Diamond/BLAST analysis, we removed nine contigs that were not assigned to Arthropoda included those assigned to Streptophyta, Basidiomycota, Ascomycota, Chordata, undefined Fungi, and Proteobacteria (Fig. 2B; Supplementary Fig. S2). We explored the results for evidence of common, managed bee pathogens, but did not find any obvious cases in the data (results not shown).

### Mitochondrial genome assembly metrics

The mitochondrial genome (NCBI accession JASKOS000000000) inferred by MitoHifi is 25,382 bp and circularized. Mitos annotation identified 12 protein-coding genes and 22 unique transfer RNAs. The genome GC content is 15.6%. We excluded the mitochondrial genome from our nuclear genome for the repeat analysis and subsequent gene annotation.

### Draft genome assembly metrics

The final draft genome assembly for *P. meconis* (ToLID iyPerMec1; https://id.tol.sanger.ac.uk/) contains 98 arthropod-specific contigs measuring a total of 326,823,531 bp (~327 Mb), or effectively the entire assembly size (Fig. 2C). The longest scaffold remains 31.8 Mb long, with an N50 length of 17.5 Mb, and an N90 length of 3.03 Mb. The final draft assembly GC content is 38.3% (Fig. 2C). The final draft nuclear genome has been uploaded to NCBI (accession JASKOS000000000; see Data Availability section).

### Chromosome identification

We found that, for both species alignments, dot plots show both contiguity and some rearrangements and/or missassemblies with *A. camellia* (Supplementary Fig. S3) and *A. mellifera* (Supplementary Fig. S4).

### Repeat and gene annotation

RepeatModeler identified repeats and subsequently masked 37.3% of the iyPerMec1 genome (Table 2). Categories of interspersed repeats included retroelements (6.07%), DNA transposons (4.38%), rolling circles (0.24%), and a large proportion (24.87%) of unclassified repeats.

**Table 2.** Repetitive elements in the iyPerMec1 genome.

Kind	Number of elements	Length occupied	Percentage of sequence
Retroelements	54,755	19,838,878 bp	6.07%
SINEs	544	75,512 bp	0.02%
Penelope	0	0 bp	0.00%
LINEs	30,469	7,797,321 bp	2.39%
CRE/SLACS	0	0 bp	0.00%
L2/CR1/Rex	11,019	2,646,616 bp	0.81%
R1/LOA/Jockey	17,172	3,958,079 bp	1.21%
R2/R4/NeSL	39	8,451 bp	0.00%
RTE/Bov-B	973	768,277 bp	0.24%
L1/CIN4	0	0 bp	0.00%
LTR elements	23,742	11,966,045 bp	3.66%
BEL/Pao	792	1,026,989 bp	0.31%
Ty1/Copia	4,577	2,142,392 bp	0.66%
Gypsy/DIRS1	9,755	6,392,493 bp	1.96%
Retroviral	0	0 bp	0.00%
DNA transposons	60,311	14,324,072 bp	4.38%
hobo-Activator	13,128	3,109,154 bp	0.95%
Tc1-IS630-Pogo	32,938	6,161,899 bp	1.89%
En-Spm	0	0 bp	0.00%
MULE-MuDR	72	19,372 bp	0.01%
PiggyBac	4,491	1,093,489 bp	0.33%
Tourist/Harbinger	1,108	231,923 bp	0.07%
Other (Mirage, P-element, Transib)	449	170,409 bp	0.05%
Rolling-circles	3,470	779,736 bp	0.24%
Unclassified	467,236	81,283,810 bp	24.87%
Total interspersed repeats		115,446,760 bp	35.33%
Small RNA	9,149	1,946,692 bp	0.60%
Satellites	70	18,217 bp	0.01%
Simple repeats	66,325	3,247,657 bp	0.99%
Low complexity	10,495	532,726 bp	0.16%

The Braker3 workflow initially annotated 23,029 protein-coding genes and 24,456 transcripts, then Interproscan provided protein annotations for 19,453 transcripts. From these transcripts with annotations, we excluded transcripts that did not have annotated start and end codons. Ultimately, our final annotation set includes 18,245 protein-coding genes and 19,433 transcripts. Aspects of this filtering approach follow previous studies in hymenopterans (e.g. the honey bee; Elsik et al. 2014).

### Phylogenetics

**Fig. 3.**
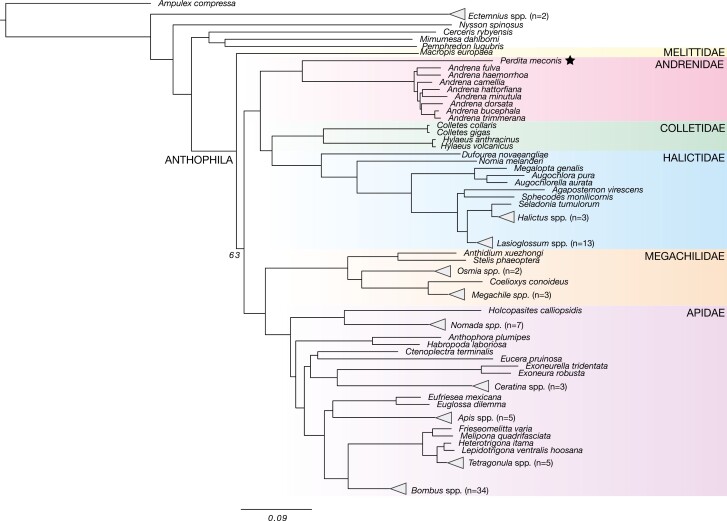
Inferred phylogeny of all publicly available Apoidea genomes based on UCE data from 119 species (1 indiv/species), including *Perdita meconis* iyPerMec1 (shown with ★), shows major bee families including Apidae, Megachilidae, Andrenidae, Melittidae, Colletidae, and Halictidae, as well as outgroup clades containing apoid wasps. Nodes with unlabeled support values are >95% ultrafast bootstrap (UFB) percentages. Scale at the bottom is the number of substitutions per site. An uncollapsed version of the phylogeny is provided in Supplementary Fig. S5.

We extracted 2,450 UCE loci from 119 genomes, including iyPerMec1 of *P. meconis*, and generated a concatenated dataset containing 2,057,031 bp of sequence data and 1,337,673 informative sites. We recovered a robust phylogeny of Apoidea that includes all bee families except Stenotritidae (endemic to Australia), and a few additional apoid wasp lineages (Fig. 3). Although the bee families Halictidae and Apidae, which contain social species, are over-represented, the majority of nodes in the tree are highly supported with 100% bootstrap support. *Perdita meconis* is resolved as the sister group to *Andrena* (family Andrenidae, subfamily Andreninae). An uncollapsed phylogeny with all 119 genomes is shown in Supplementary Fig. S5.

## Discussion

We generated a high-quality reference genome for a bee species of conservation concern, *P. meconis*, the Mojave poppy bee. The genome contains a high proportion of complete Hymenopteran BUSCO genes and has an N50 of 17.5 Mb. Furthermore, we were able to identify and annotate 18,245 genes, which will provide a valuable resource for gene-related and/or functional studies. Many of these metrics are similar to published values for other bee species (Table 3; citations therein), although the number of annotated coding genes is on the upper end. We hope that additional samples of *P. meconis* may be collected and used to generate RNAseq data for further improvements in annotation. The high quality of this genome is especially impressive given both the very small size of the organism and the fact that the specimen was not preserved under ideal circumstances (i.e. not snap frozen, stored at −20 °C rather than −80 °C). This outcome builds upon previous studies that generated reference genomes from single, albeit larger, insect specimens (Kingan et al. 2019; Koch et al. 2023), and suggests that insect conservation genomics may benefit greatly from further improvements in sequencing chemistries and technologies.

**Table 3.** Genome assembly statistics of *Perdita meconis* compared with other bee genomes.

Species	Genome size (Mb)	Number of contigs	Contig N50 (Mb)	Assembly Level	GC content	Number of protein-coding genes	Reference
*Perdita meconis*	327	107	17.5	Contig	38.30%	18,245	Present study
*Andrena camellia*	340.73	131	17.2	Chromosome	42.25%	11,258	Zhao et al. (2023)
*Andrena minutala*	380	569	12.7	Chromosome	40.30%	10,936	Falk et al. (2022a)
*Bombus affinis*	365.1	858	12.3	Chromosome	37.40%	10,718	Koch et al. (2023)
*Apis mellifera*	225.2	227	5.4	Chromosome	32.50%	9,935	Wallberg et al. (2019)
*Colletes collaris*	374.75	374	8.96	Contig	41.42%	20,399	Ferrari et al. (2023)
*Macropis europaea*	546.8	2,580	8.4	Chromosome	38%	n/a	Falk et al. (2022b)
*Nomia melanderi*	325.5	108,529	28.9	Scaffold	40%	9,924	Kapheim et al. (2019)
*Eucera pruinosa*	409.1	797	10.6	Scaffold	37%	8093	Pope et al. (2023)

While most contemporary genome assemblies include techniques to elevate contig assemblies to chromosomes, we did not include HiC sequencing in this study due to difficulty in obtaining samples stored to retain intact nuclei. Despite this, the genome is highly contiguous and can still be used for comparative genomic studies as many contigs are likely near chromosomal. The number of chromosomes in bees ranges from 5 to 38, with a mean of 17 (NCBI metadata, accessed 11 November 2023). Thus, our assembly with 22 contigs accounting for 90% of the total length is within the observed range for bees. The genome-genome alignment plots showed less contiguity than we expected, which is likely due to the phylogenetic distance between the new genome and the references. Even though *Andrena* is in the same family, it belongs to a separate subfamily that diverged ~90 million years ago (Bossert et al. 2022). Bees are ~110 million years old, so the phylogenetic distances among *Perdita*, *Andrena,* and *Apis* are all large. Future field efforts will aim to get additional, flash-frozen samples for high-molecular-weight DNA extraction and subsequent genome scaffolding.

Using RepeatModeler and RepeatMasker, we identified almost 40% of the genome as containing repetitive elements. This proportion seems to fall within the published ranges for insects (Sproul et al. 2023), including a recent genome assembly of *A. camellia* that contained 44.65% repeats (Zhao et al. 2023). Unfortunately, there is a lack of information on the classification of repeat elements in insects that are not model organisms (e.g. *Drosophila*, Sproul et al. 2023).

Among the bees, of which there are more than 20,000 species globally, the majority of genomes have been generated from only a small set of phylogenetically clustered social species within the families Apidae (honey bees, bumble bees) and Halictidae (sweat bees). This bias reflects the challenges and high costs of generating high-quality reference genomes as well as the general interest within the research community in understanding the evolution of sociality and in targeting species of agricultural and economic importance (Woodard et al. 2020).

Andrenidae is a highly diverse family of bees, yet relatively few genomes have been sequenced thus far. Our high-quality genome for *P. meconis* represents the first publicly available *Perdita* and the first representative of the large subfamily Panurginae. All other genomes of bees within Andrenidae are from the genus *Andrena* (e.g. Zhao et al. 2023; Falk et al. 2023a). Given the threatened nature of *P. meconis* and susceptibility to threat of other *Perdita* species, this genome will provide an annotated, high-quality reference for future conservation genomics studies aimed at understanding and preserving *Perdita* species. For example, aided by the reference genome, one might examine genetic structure, gene flow, and dispersal among populations, determine the effective population size, find evidence of inbreeding, and determine which populations might be most at risk. These potential research areas, as well as designing assays for noninvasive environmental DNA monitoring (e.g. Laramie et al. 2015; Thomsen and Sigsaard 2019), can provide insight for future conservation decisions for this species of concern.

## Supplementary material

Supplementary material is available at *Journal of Heredity* Journal online.

esad076_suppl_Supplementary_Figures

## Data Availability

We have deposited the primary data underlying these analyses as follows: DNA sequences: NCBI SRA (#SRR25939583) as part of the Beenome100 umbrella project (#PRJNA923301). Final draft genome assembly uploaded as a Whole-Genome Shotgun project at DDBJ/ENA/GenBank under the accession JASKOS000000000.
