# Supplementary material for: Reference genome for the Mojave poppy bee (Perdita meconis), a specialist pollinator of conservation concern
Source: J Hered. 2023 Dec 13;115(4):470–9. doi: 10.1093/jhered/esad076 (PMC11235129; doi:10.1093/jhered/esad076)
Supplement: esad076_suppl_Supplementary_Figures [file esad076_suppl_supplementary_figures.zip › esad076_suppl_Supplementary_Figures_S1-S5/esad076_suppl_Supplementary_Figures_S1-S5.docx]

**Supplemental Tables and Figures**

**Figure S1.**


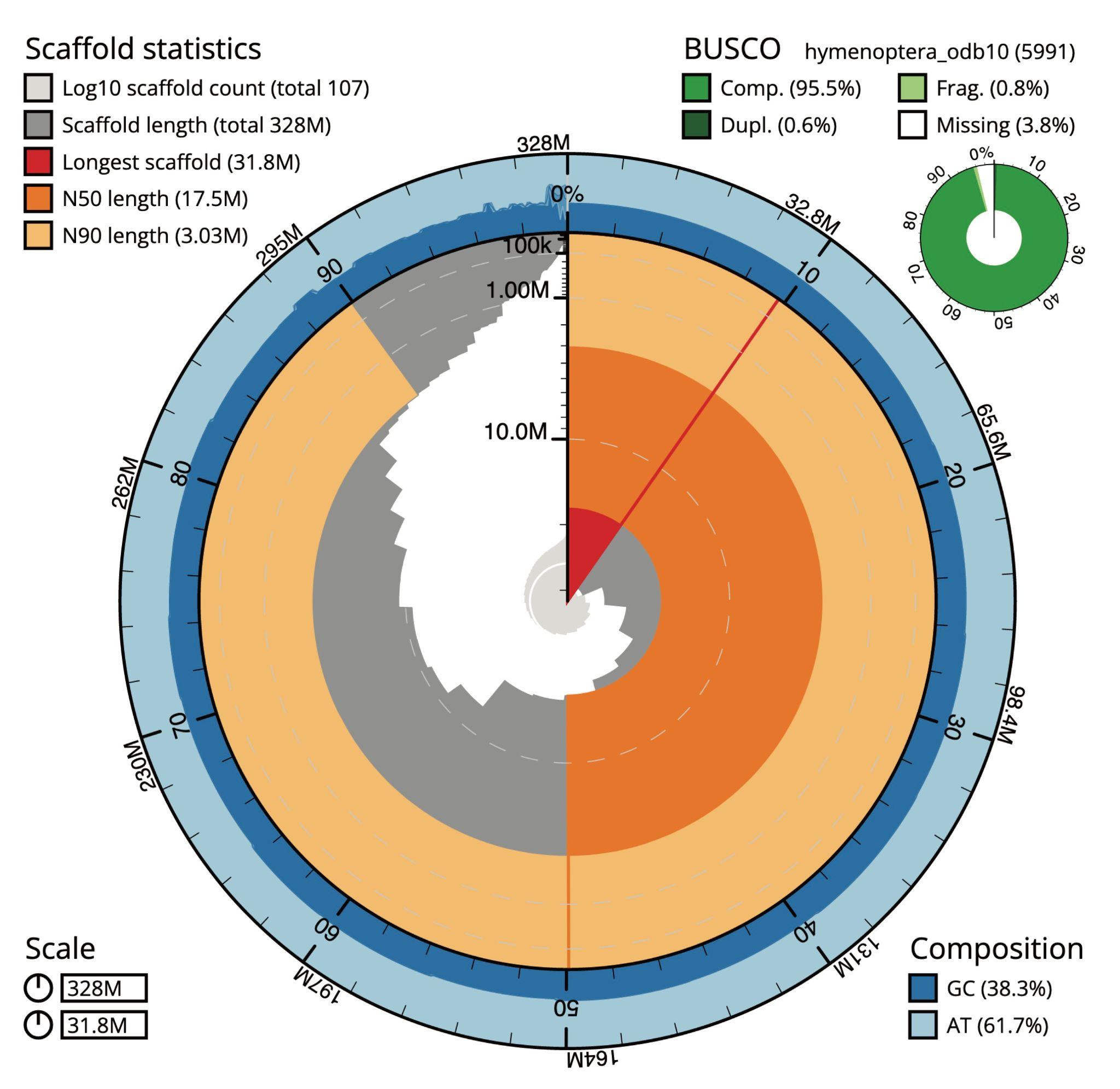


**Figure S1.** Snail plot of Mojave poppy bee initial genome assembly before removal of non-Arthropod contigs. The main plot is divided into 1,000 size-ordered bins around the circumference with each bin representing 0.1% of the 327,936,261 bp assembly. The distribution of sequence lengths is shown in dark grey with the plot radius scaled to the longest sequence present in the assembly (31,838,323 bp, shown in red).. Orange and pale-orange arcs show the N50 and N90 sequence lengths (17,524,680 and 3,032,719 bp), respectively. The pale grey spiral shows the cumulative sequence count on a log scale with white scale lines showing successive orders of magnitude. The blue and pale-blue area around the outside of the plot shows the distribution of GC, AT and N percentages in the same bins as the inner plot. A summary of complete, fragmented, duplicated and missing BUSCO genes in the hymenoptera_odb10 set is shown in the top right.

**Figure S2.**


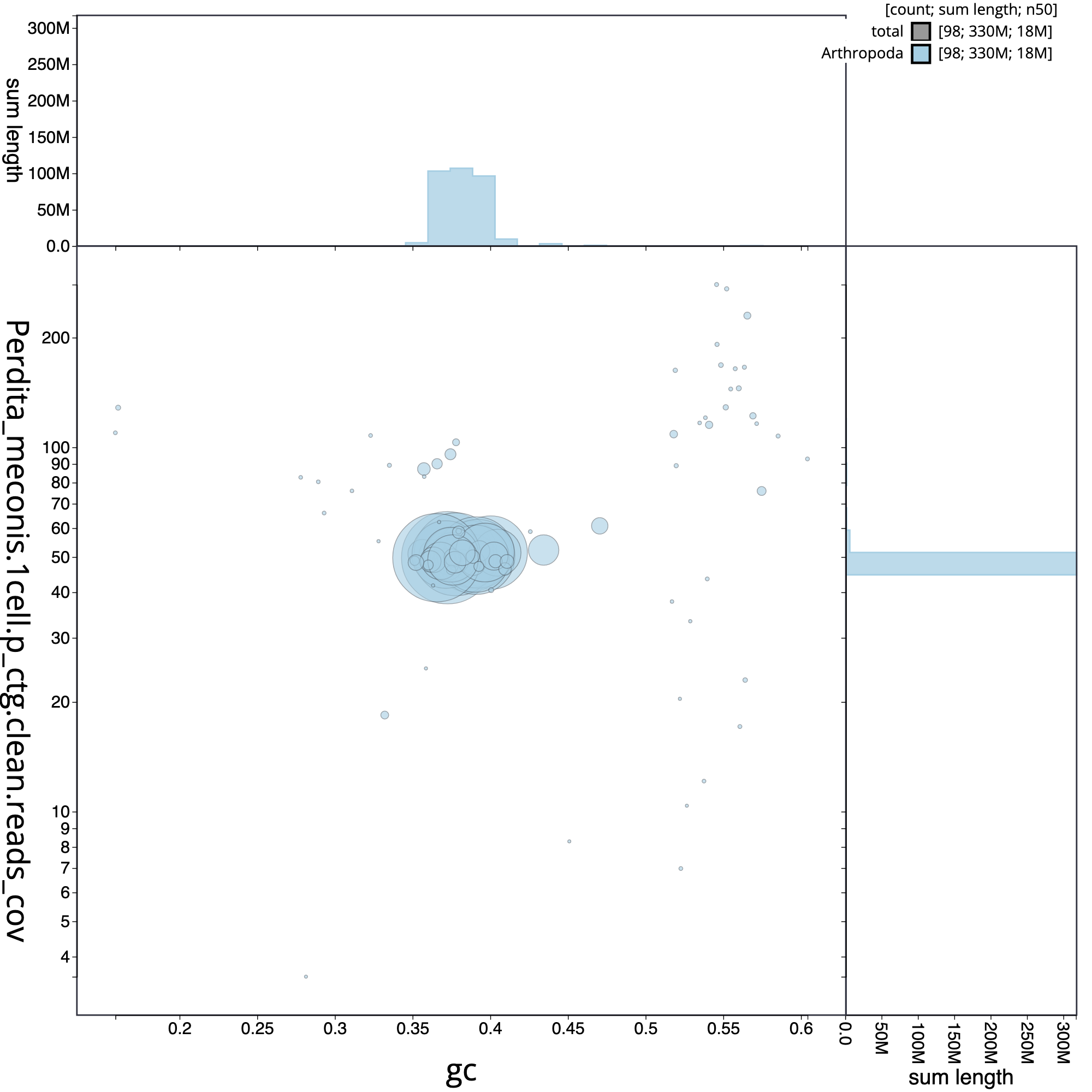


**Figure S2.** Blob plot for Mojave poppy bee genome assembly after removing all non-Arthropod contigs. Each blob represents a contig, colored according to the phylum with the best match in BLAST.


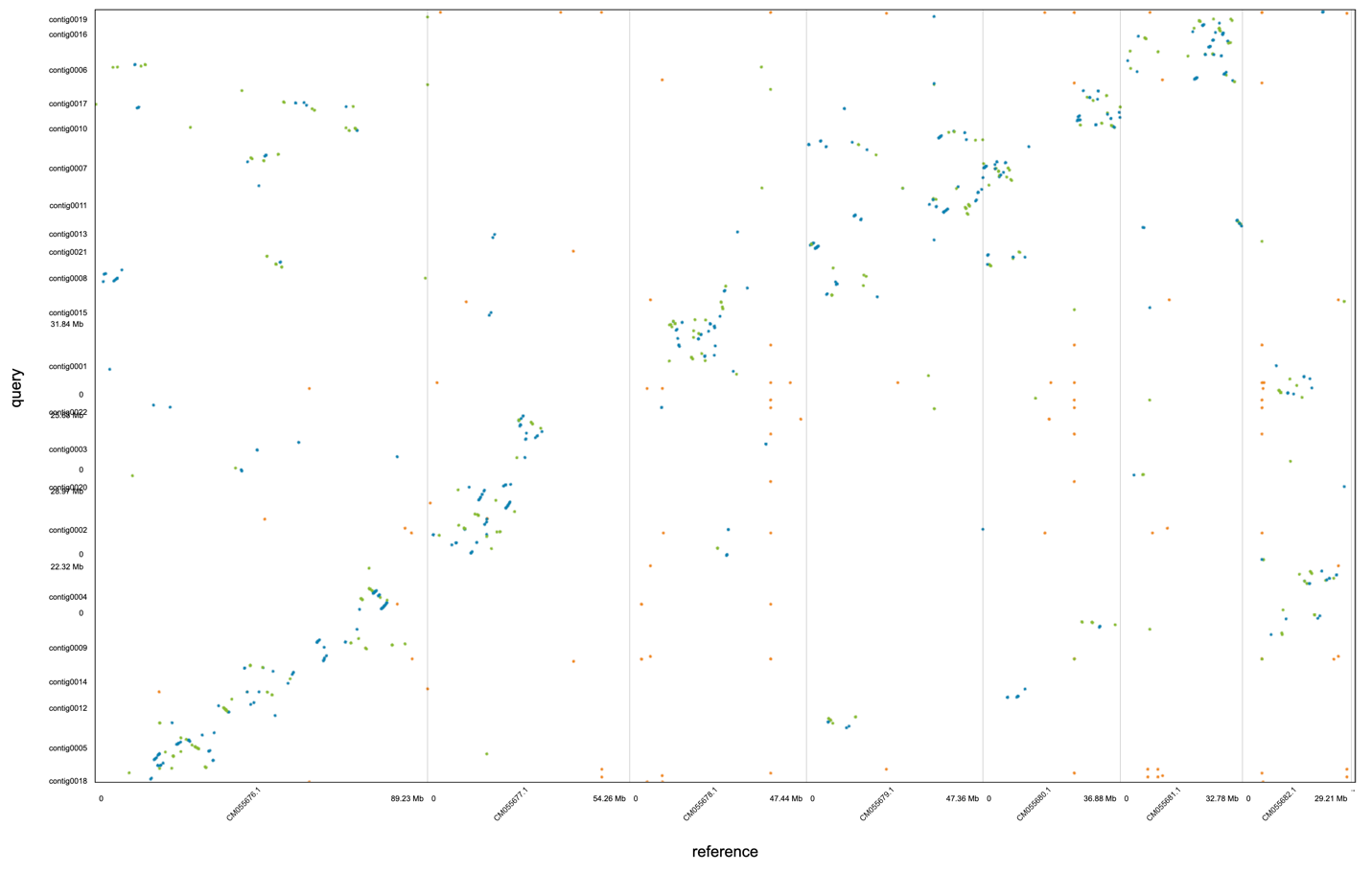


**Figure S3**. Dot plot for alignment of *Andrena camellia* (“reference”) to *Perdita meconis* (“query”). *A. camellia* chromosomes are ordered from largest to smallest. Blue dots represent unique forward alignments, green dots represent unique reverse alignments, and orange dots represent repetitive alignments.


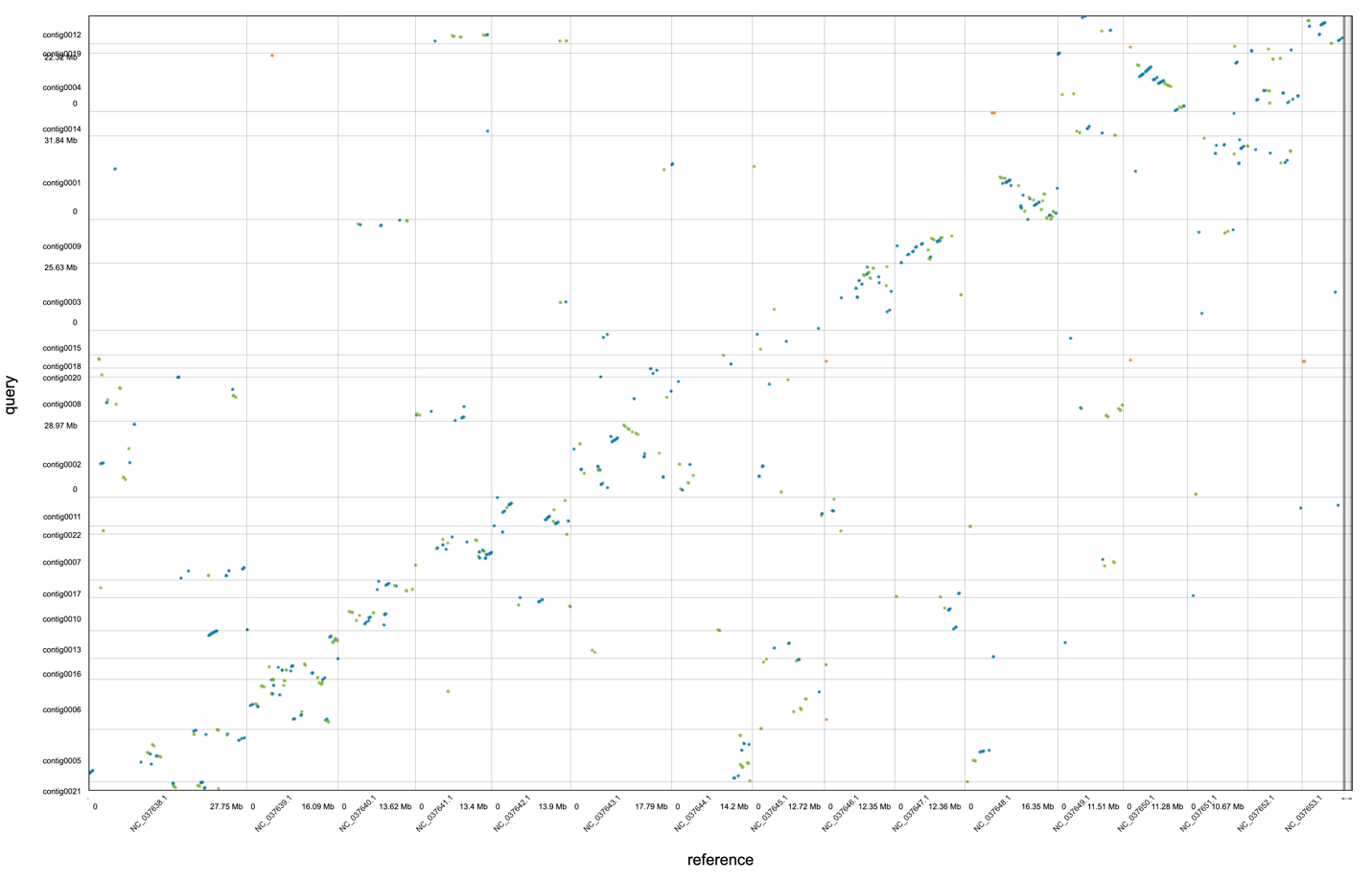


**Figure S4**. Dot plot for alignment of *Apis mellifera* (“reference”) to *Perdita meconis* (“query”). *A. mellifera* chromosomes are ordered from largest to smallest. Blue dots represent unique forward alignments, green dots represent unique reverse alignments, and orange dots represent repetitive alignments.


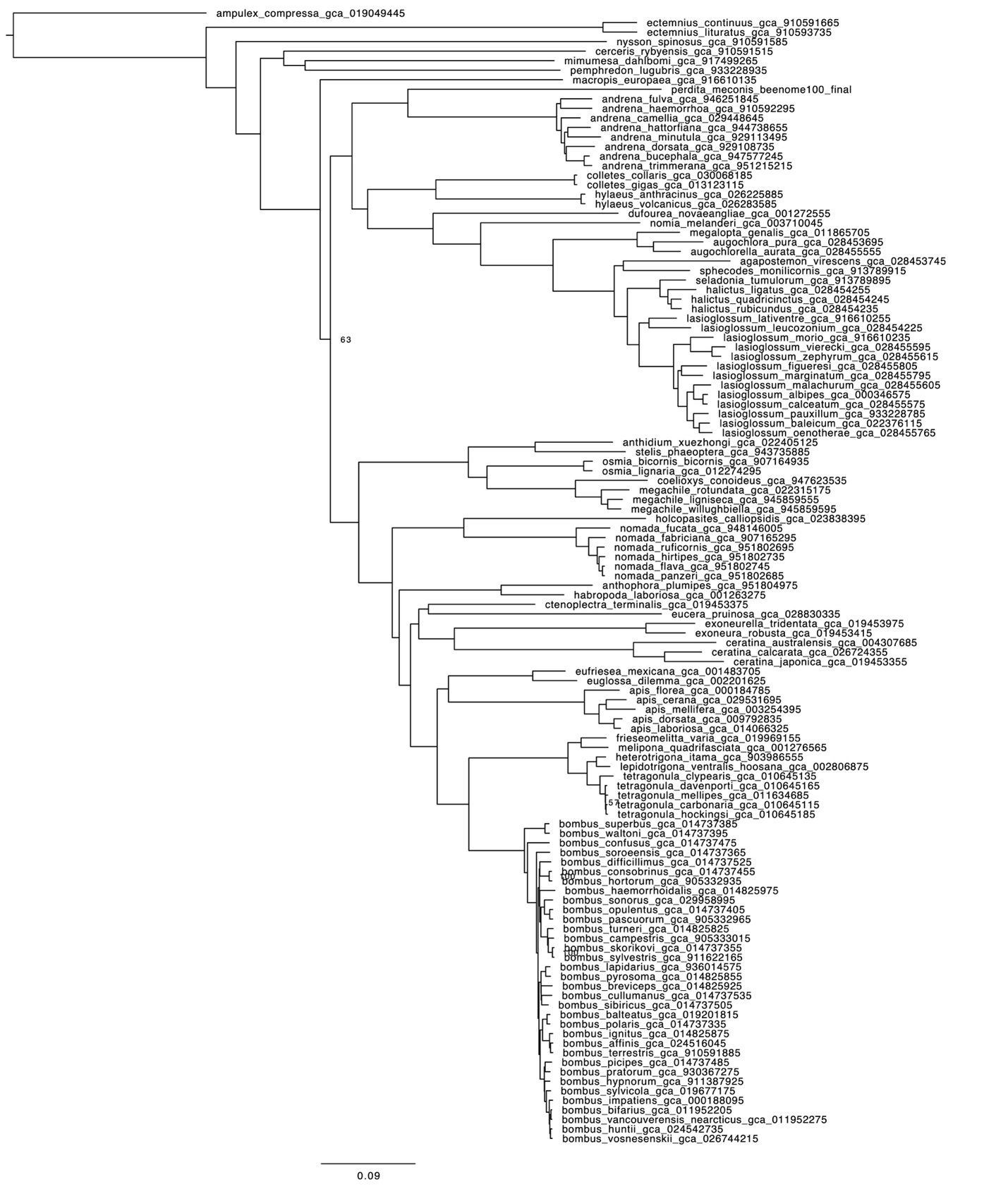


**Figure S5.** Inferred phylogeny of all publicly available Apoidea based on UCE data from 119 species (1 indiv/species), including iyPerMec1, shows major bee families including Apidae, Megachilidae, Andrenidae, Melittidae, and Colletidae, as well as outgroup clades containing wasps. Nodes with unlabeled support values are >95%. Scale at the bottom is the number of substitutions per site.
