# Supplementary figures and images for: Reference genome for the Mojave poppy bee (Perdita meconis), a specialist pollinator of conservation concern
Source: J Hered. 2023 Dec 13;115(4):470–9. doi: 10.1093/jhered/esad076 (PMC11235129; doi:10.1093/jhered/esad076)

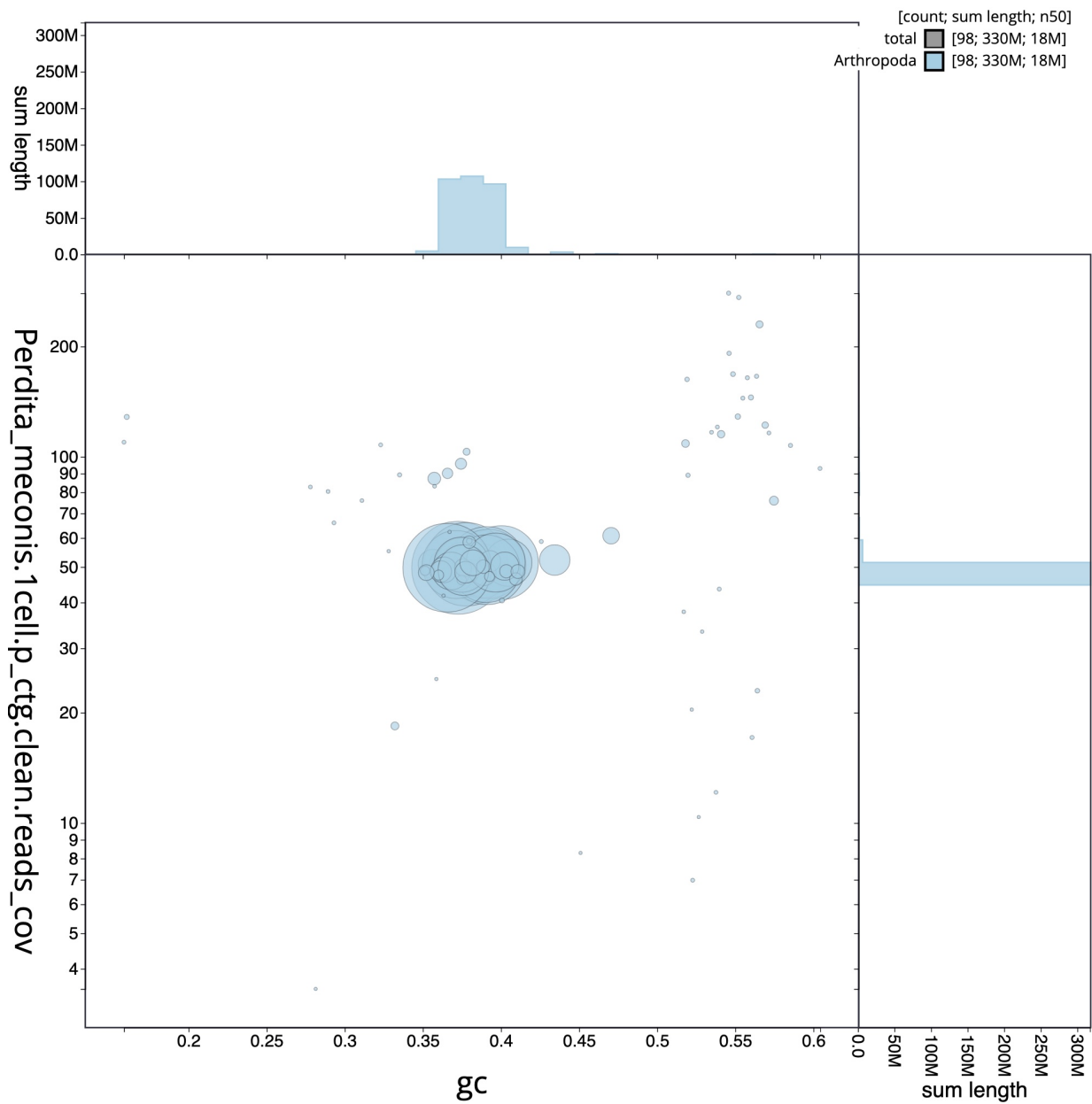

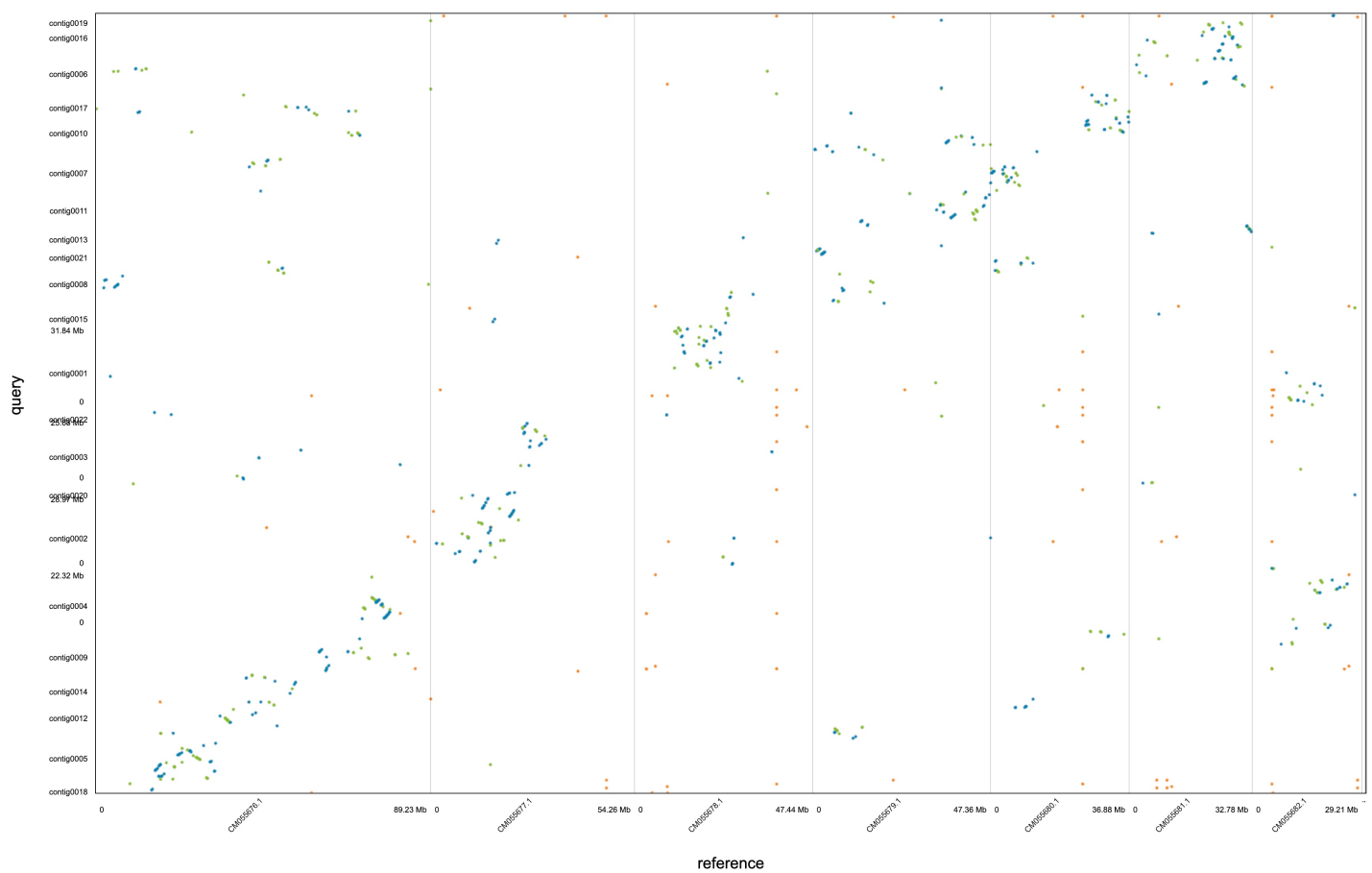

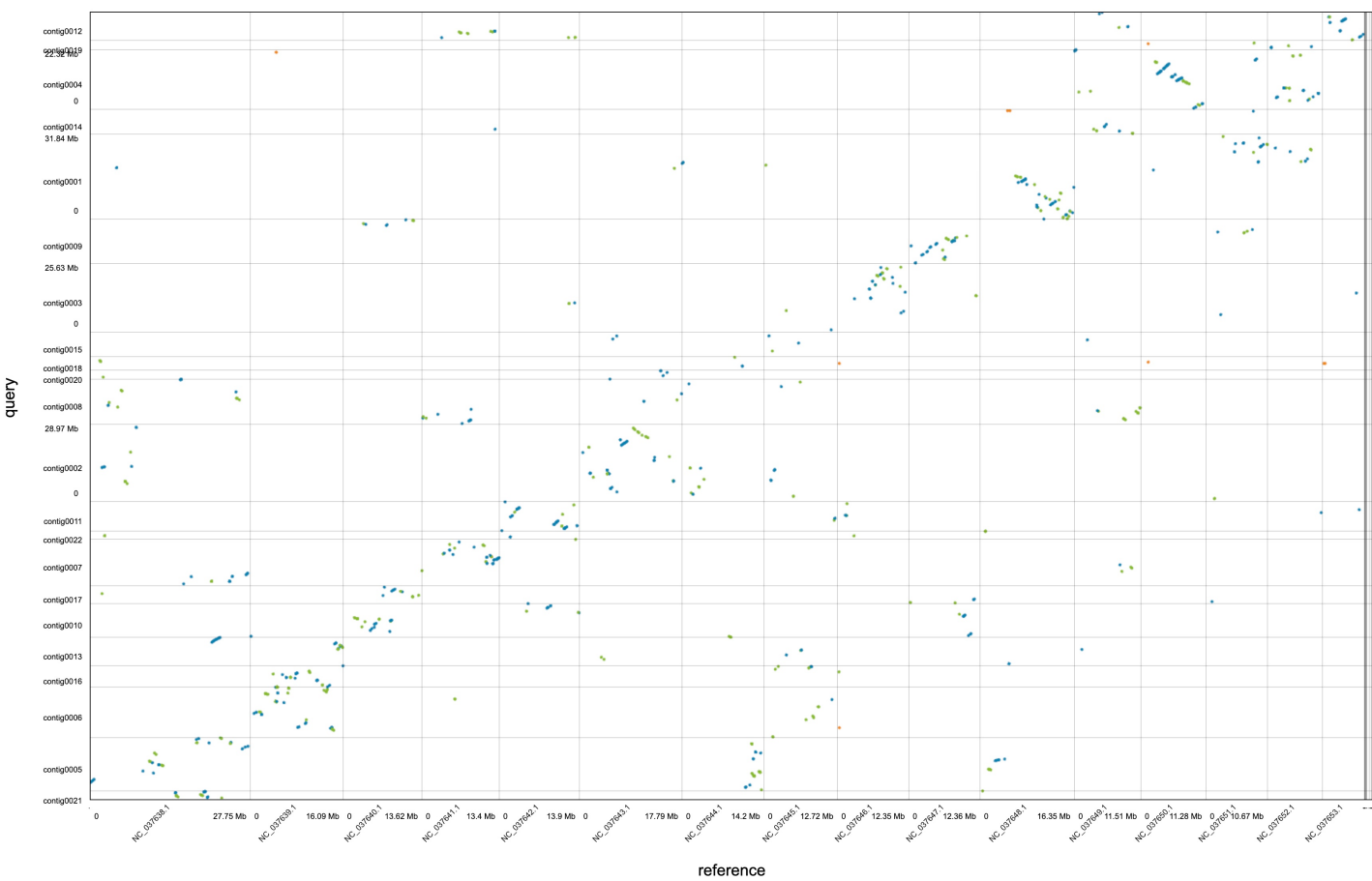

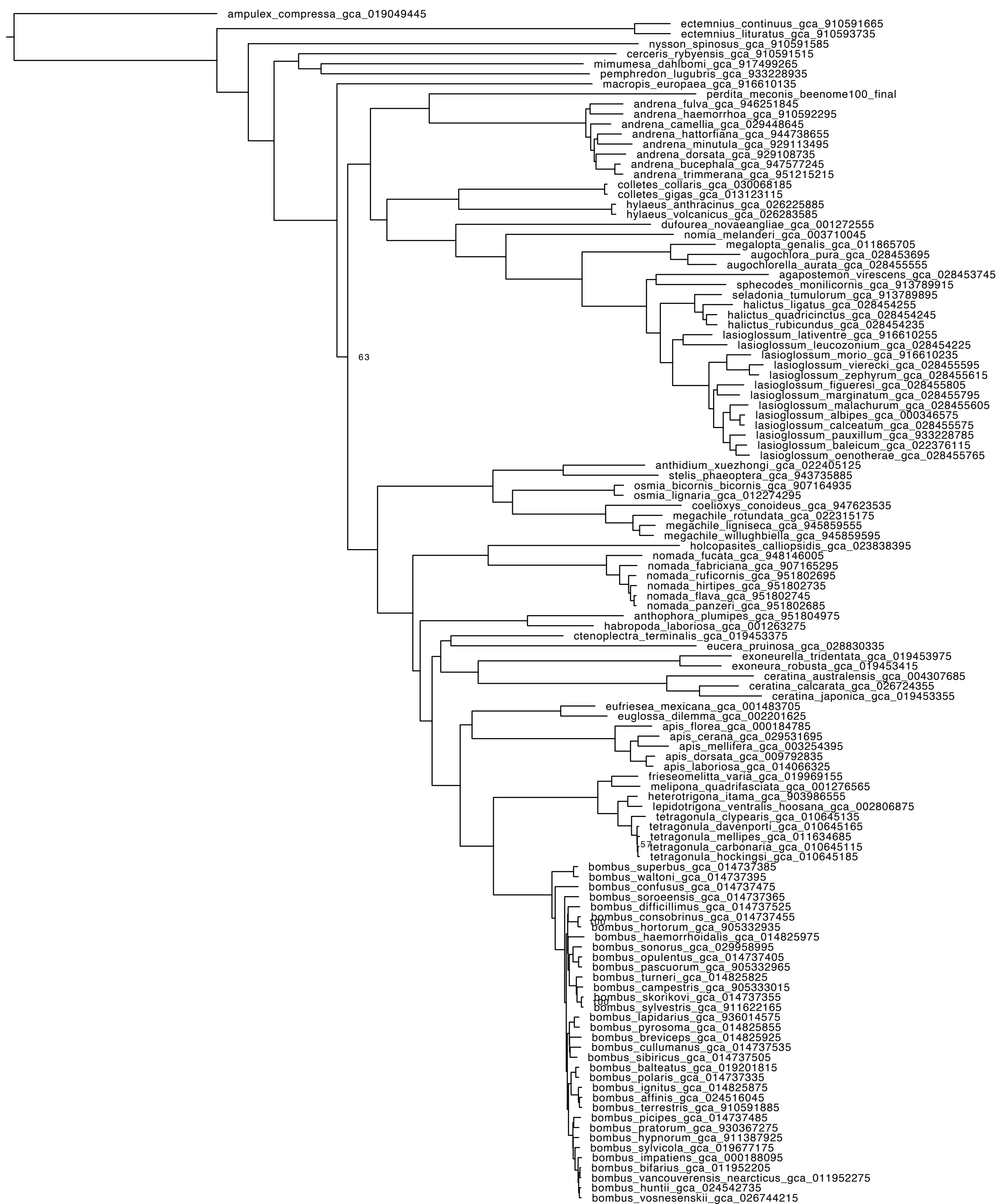

0.09

Supplement: esad076_suppl_Supplementary_Figures [file esad076_suppl_supplementary_figures.zip › esad076_suppl_Supplementary_Figures_S1-S5/esad076_suppl_Supplementary_Figures_S1-S5.pdf]
